# Metabolite Profiles Correlate Closely with Neurobehavioral Function in Experimental Spinal Cord Injury in Rats

**DOI:** 10.1371/journal.pone.0043152

**Published:** 2012-08-13

**Authors:** Yusuke Fujieda, Shinya Ueno, Ryoko Ogino, Mariko Kuroda, Thomas J. Jönsson, Lining Guo, Takeshi Bamba, Eiichiro Fukusaki

**Affiliations:** 1 Department of Biotechnology, Graduate School of Engineering, Osaka University, Suita, Japan; 2 Asubio Pharma Co., Limited, Kobe, Japan; 3 Metabolon Inc., Durham, North Carolina, United States of America; Hertie Institute for Clinical Brain Research, University of Tuebingen, Germany

## Abstract

Traumatic spinal cord injury (SCI) results in direct physical damage and the generation of local factors contributing to secondary pathogenesis. Untargeted metabolomic profiling was used to uncover metabolic changes and to identify relationships between metabolites and neurobehavioral functions in the spinal cord after injury in rats. In the early metabolic phase, neuronal signaling, stress, and inflammation-associated metabolites were strongly altered. A dynamic inflammatory response consisting of elevated levels of prostaglandin E2 and palmitoyl ethanolamide as well as pro- and anti-inflammatory polyunsaturated fatty acids was observed. *N-*acetyl-aspartyl-glutamate (NAAG) and *N-*acetyl-aspartate (NAA) were significantly decreased possibly reflecting neuronal cell death. A second metabolic phase was also seen, consistent with membrane remodeling and antioxidant defense response. These metabolomic changes were consistent with the pathology and progression of SCI. Several metabolites, including NAA, NAAG, and the ω-3 fatty acids docosapentaenoate and docosahexaenoate correlated greatly with the established Basso, Beattie and Bresnahan locomotive score (BBB score). Our findings suggest the possibility of a biochemical basis for BBB score and illustrate that metabolites may correlate with neurobehavior. In particular the NAA level in the spinal cord might provide a meaningful biomarker that could help to determine the degree of injury severity and prognosticate neurologic recovery.

## Introduction

Spinal cord injury (SCI) often leads to severe disability due to permanent neurological impairment. The annual incidence of SCI lies between 10 to 80 persons per million and well over 30% of cases have some degree of tetraplegia [1], [2]. The life expectancy of patients with acute SCI is dramatically and progressively shortened in relation to the degree of spinal cord injury and neurological deficit [3]. There are few therapeutic interventions that limit the extent of tissue damage following SCI [1]. Consequently SCI is a catastrophic medical condition which dramatically reduces the patient’s quality of life and imposes disproportionately large economic and social costs on affected individuals and society in general [2].

The mechanistic insult to the spinal cord induces a variety of parallel pathophysiological processes [4]–[6]. In the early phase axons are disrupted, neural cell death occurs, and blood vessels are damaged, resulting in hemorrhage that exacerbates the ischemic neural injury. In the secondary phase necrotic death, electrolytic shifts, and edema continue. Cytotoxic levels of tissue debris, neurotransmitters, and reactive oxygen species are formed that promote an inflammatory response. Local tissue remodeling, neuroprotective and regenerative processes are also initiated that contribute to limited spontaneous improvement.

Accurate behavioral evaluation of SCI animals is an important tool for evaluating the therapeutic efficacy of pharmaceutical drugs. The Basso-Beattie-Bresnahan (BBB) locomotor rating scale is widely used to test behavioral consequences of spinal cord injury in rodents [7]. In this test joint movements, paw placement, weight support, forelimb and hindlimb coordination are judged by experienced examiners according to the 21-point BBB locomotion scale. However, a simple and reliable quantitative chemical assay that readily could be adopted in a laboratory to monitor the outcome of SCI has yet to be developed. Furthermore, finding the biomarkers that could help to determine the degree of injury severity and to prognosticate neurologic recovery is one of the major challenges in management of SCI [8], [9]. Identification of reliable, low invasive biomarkers would be helpful for the clinician and patients in the choice of potential treatments.

Metabolomics is a new approach that involves the determination of changes in the levels of endogenous or exogenous metabolites in biological samples, owing to physiological stimuli or genetic modification [10], [11]. The power of metabolomics lies in the global determination of metabolites, or patterns of biomarkers that increase or decrease as the result of a particular disease or injury. Here we have applied mass spectrometry (MS) based metabolomic technology in order to investigate the global metabolomic impact of SCI and to identify metabolites that could potentially be used to assess behavioral consequences of spinal cord injury.

## Materials and Methods

### Materials and Reagents

NAA and NAA-1,2,3,4-^13^C4 (NAA-^13^C4) were from Wako Pure Chemicals (Japan) and SIGMA-Aldrich (St. Louis, MO), respectively. *N-*Methyl-*N-*trimethylsilyl-trifluoroacetamide (MSTFA) was from GL Science (Japan). Other reagents and materials were of analytical or special grade.

### Animals Preparation

The protocol was approved by the Ethics Committee for Animal Experiments of Asubio Pharma CO., LTD. (Permit Number: 9–47) and carried out in strict accordance with the Guideline for Animal Experiments of the laboratories. All surgery was performed under pentobarbital sodium, and all efforts were made to minimize suffering.

### Metabolomic Analysis in Rat Spinal Cord Tissues after SCI or Sham Operation

Eight week old female Sprague-Dawley rats were purchased (Japan SLC, Inc., Japan), and used shortly thereafter at weights ranging from 169 to 212 g. They were divided into six groups: Two-, eleven- and thirty-days after injury and day-matched sham controls (without injury to the cord). The number of animals in each group was 7 to 8. The following operation was done after animals were anesthetized with intraperitoneal injection of 50 mg/kg pentobarbital sodium (Somnopentyl, Kyoritsu Seiyaku Co., Japan). A laminectomy was performed at the ninth thoracic vertebra (T9), and the exposed spinal site was kept just under the device. A 200-kdyn force was delivered to the exposed cord to produce a severe level of injury using an Infinite Horizon Impactor (Precision Systems and Instrumentation, LLC, Lexington, KY). Tonic extension of the hindlimbs by the spinal cord injury was checked. The animals were allowed to recover in warmed cages and received 0.1% of Bactramin (Bactramin inj., a mixture of Trimethoprim and Sulfamethoxazole (16 mg and 80 mg/mL), Chugai Pharmaceutical Co., Ltd., Japan) in drinking water for 10 days. The injured animals’ bladders were manually expressed once daily for 10 days. Animals had free access to food and water. Only rats that showed complete paralysis (BBB score  = 0) the day after the operation were used.

The motor function of the hindlimbs was evaluated according to the Basso-Beattie-Bresnahan locomotor scale [7] just before sampling. The BBB score ranged from 0 (complete paralysis) to 21 (normal gait). Two observers measured motor dysfunction of the right and left hindlimbs respectively and their mean value was employed as individual animal data.

After the behavioral assessment, the rats were deeply anesthetized by pentobarbital sodium and 2.5 cm in length of spinal cord, centered at the lesion epicenter, were dissected to obtain enough amounts of tissue samples. Spinal cord tissues were immediately frozen by liquid nitrogen and stored under −70 degree Celsius until measurement.

Metabolomic profiling was performed as described previously [12], [13]. Samples ranging from 112–221 mg were homogenized by bead beating on a GenoGrinder 2000 in the presence of a four-fold ratio of water (w/v). Metabolites were extracted from a 100 µL quantity of homogenate by the addition of cold methanol. The precipitated extract was split into four aliquots and dried under nitrogen and then in vacuo. The samples were resuspended in platform specific solutions before they were applied into the instruments. The untargeted metabolomic profiling platform employed for this analysis was based on a combination of three independent platforms: ultrahigh performance liquid chromatography/tandem mass spectrometry (UHPLC/MS/MS) [12] optimized for basic species, UHPLC/MS/MS optimized for acidic species, and gas chromatography/mass spectrometry (GC/MS) [13]. Metabolites were identified by matching the ions’ chromatographic retention index and mass spectral fragmentation signatures with reference library entries created from authentic standard metabolites. For ions that were not covered by the standards, additional library entries were added based on their unique retention time and ion signatures. Peak ion counts for each compound in each sample were used for statistical analysis, resulting in the comparisons of relative concentrations. A given compound was reported from only one of the three platforms.

### NAA Quantification in Rat Spinal Cord after SCI or Sham Operation

Eight weeks old female Sprague-Dawley rats were purchased (Japan SLC, Inc.), and used within a week at 178 to 209 g. We chose the three injury forces to get animals with a variety of BBB score. They were divided into four groups of: Sham controls (n = 2) and 100-kdyn (mild: n = 6), 150-kdyn (moderate: n = 6) and 200-kdyn (severe: n = 6) injured group. Animals were prepared as described earlier and 100, 150 or 200-kdyn injury inducing forces were delivered to the exposed cord to produce a wide variety of symptoms. Only animals that showed complete paralysis (BBB score  = 0) the day after the operation were used. The motor function of the hindlimbs was evaluated according to BBB scale just before the sampling (at 32–34 days post injury). One animal in 100-kdyn injured group showed no recovery in BBB score and was excluded. After the behavioral assessment, the rats were deeply anesthetized and spinal cord tissues around epicenter were collected as described in the previous section.

Spinal cord tissues ranging from 118 to 187 mg were homogenized in the presence of a nine-fold ratio of water (w/v) and the water layer was extracted from each homogenate by modified Bligh-Dyer method [14]. For the stable isotope dilution, NAA-^13^C was added to the specimens as an internal standard. Samples were converted to their trimethylsilyl derivatives (TMS) prior to GC–MS analysis by using MSTFA. An Agilent7000 GC-MS/MS system was used for the quantification of NAA concentration in spinal cord tissue. Scanning was in the multiple-reaction monitoring (MRM) using mass-to-charge transitions of 274>184 for NAA and 277>187 for NAA-^13^C.

### Statistical Analysis

Welch’s two sample *t*-test was used to compare data obtained from SCI rats at days 2, 11 and 30 and day-matched sham. False discovery rates (FDR) were computed because of the multiple comparisons using the q-value method [15]. The q-values were estimated with the R-package [16]. Correlation coefficients were calculated by Microsoft Excel and PCA analysis was performed using SIMCA-P+ software (Ver. 12.0, Umetrics Inc, Umeå, Sweden).

## Results

### Global Metabolomic Changes Following SCI

To understand the metabolic impact of severe SCI an untargeted metabolomic profiling approach was taken to assess the chemical milieu of the spinal cord. 405 metabolites were detected of which 283 matched known structures in our chemical reference library. Welch’s two sample *t*-test was used to analyze the differences between the sham and SCI treated rats at days 2, 11 and 30. The metabolites matched with known chemical structures and their statistical comparisons by *t-* test are listed in Dataset S1.

Pathophysiological changes of traumatic acute spinal cord injury can be divided into the three phases which are acute, recovery and plateau phase. Therefore, we selected three sampling points which are two- (acute), eleven- (recovery) and thirty-days (plateau) after SCI.

**Figure 1:**
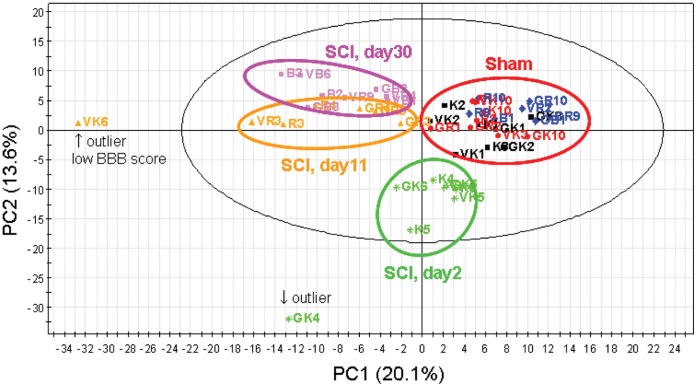
PCA Score plot of rat spinal cord samples in SCI or Sham operated animals with identification number. Black ellipse in the score plot illustrates the 95% confidence regions. PCA score plot displaying the distinct dissimilarities between Sham animals and SCI animals, and also suggested the time-dependent changes in metabolite profiles after SCI operation. There are two outlier in PCA score plot; one of them (animal No. VK6) in SCI day11 group showed low BBB score.

SCI induced dramatic metabolic changes with more than 25% of the profiled metabolites significantly altered. Clear metabolic separation between the sham and SCI animals was observed by principal component analysis (PCA) (Figure 1). Moreover, time-dependent changes in metabolite profiles after SCI operation were also observed reflecting the dramatic longitudinal effect of the damage. Typical metabolites contributing to the separation in PCA score plot are listed in Table 1.

**Table 1:** Summary of altered spinal cord metabolites in the primary and secondary phase when contrasting SCI and sham treated rats.

Earlymetabolic phase	SCI vs. Sham						
Metabolite	Fold change			p-value			
*Lipid Mediator*	Day 2	Day 11	Day 30	Day 2	Day 11		Day 30
oleic ethanolamide	**7.86**	1.15	1.06	0.00	0.32		0.51
palmitoyl ethanolamide	**7.09**	1.06	1.04	<0.01	0.69		0.70
prostaglandin E2	**1.32**	1.08	NA	0.04	0.18		–
*Polyamine*							
ornithine	**1.77**	0.96	0.79	0.02	0.60		0.30
putrescine	**2.39**	**2.21**	1.31	<0.01	<0.01		0.14
spermidine	0.94	1.04	0.96	0.44	0.78		0.63
5-methylthioadenosine	0.92	0.95	0.87	0.37	0.53		0.10
*Neurotransmitters*							
glutamate	**0.78**	**0.90**	**0.87**	<0.01	0.01		<0.01
glutamine	**0.94**	1.04	**1.10**	0.05	0.32		0.02
* N-*acetyl-aspartyl-glutamate	0.86	**0.80**	**0.74**	0.06	<0.01		<0.01
gamma-aminobutyrate	0.86	0.90	**0.78**	0.06	0.44		0.01
aspartate	**0.65**	**0.85**	**0.91**	0.00	0.00		0.04
* N-*acetylaspartate	0.83	**0.63**	**0.54**	0.31	0.00		<0.01
**Secondary** **metabolic phase**	**SCI vs. Sham**						
**Metabolite**	**Fold change**			**p-value**			
***Membrane remodeling***	**Day 2**	**Day 11**	**Day 30**	**Day 2**	**Day 11**		**Day 30**
linolenate (18∶3n3 or 6)	**1.45**	1.87	1.21	0.01	0.24		0.12
dihomo-linolenate (20∶3n3 or n6)	0.91	**1.60**	**1.74**	0.37	0.00		<0.01
eicosapentaenoate (20∶5n3)	**1.41**	**2.25**	**2.47**	0.01	<0.01		<0.01
docosapentaenoate (22∶5n3)	**1.94**	**3.02**	**2.87**	<0.01	<0.01		<0.01
docosapentaenoate (22∶5n6)	**1.61**	**2.84**	**4.95**	0.00	0.00		<0.01
docosahexaenoate (22∶6n3)	**1.89**	**2.00**	**2.19**	0.01	<0.01		<0.01
glycerophosphorylcholine	0.92	**1.71**	**1.79**	0.39	<0.01		<0.01
docosadienoate (22∶2n6)	**1.28**	**2.31**	**2.14**	0.04	<0.01		<0.01
docosatrienoate (22∶3n3)	**1.79**	**3.34**	**2.79**	0.00	<0.01		<0.01
adrenate (22∶4n6)	**1.25**	**2.01**	**1.94**	0.03	<0.01		<0.01
1-palmitoyl-GPI	1.00	**1.37**	**1.57**	0.95	0.00		<0.01
1-stearoyl-GPI	1.12	**1.19**	**1.28**	0.39	0.05		<0.01
1-arachidonoyl-GPI	1.04	**1.84**	**1.66**	0.68	0.01		0.02
1-oleoyl-GPS	**2.24**	**2.24**	**2.76**	<0.01	<0.01		<0.01
2-oleoyl-GPS	1.20	**1.72**	**2.11**	0.25	0.02		<0.01
ethanolamine	0.97	**1.71**	1.17	0.60	0.02		0.23
phosphoethanolamine	0.79	**1.79**	**1.38**	0.05	<0.01		<0.01
*Antioxidant defense*							
ascorbate	**0.61**	**1.42**	1.11	<0.01	0.01		0.19
glutathione, (GSSG)	0.85	**3.79**	1.68	0.77	0.00		0.20
alpha-tocopherol	1.02	**1.30**	**1.29**	0.72	0.02		0.03
ergothioneine	1.07	**1.42**	**1.25**	0.34	<0.01		0.01

Statistically significant changes are in bold (p<0.05, Welch’s two sample t-test).

GPI refers to glycerophosphoinositol and GPS refers to glycerophosphoserine.

Close examination of the data revealed two metabolic phases. In the early metabolic phase (day 2) lipid mediators, polyamines and neurotransmitter-related compounds were strongly altered (Table 1). An increase in lipid mediators, oleic ethanolamide (OEA), palmitoyl ethanolamide (PEA) and prostaglandin E2 (PGE_2_) were observed at day 2. The subsequent return to basal levels indicated that their elevation was a direct response to the injury. Polyamines were also strongly affected. In particular, putrescine was greatly elevated by day two and returned to basal level after day 11 consistent with reports that ornithine decarboxylase, the rate limiting enzyme in polyamine synthesis, is strongly activated in response to stress [17], [18]. This activation may indicate both secondary pathogenesis and induction of neuroprotective response. Dramatic changes in neurotransmitters and several of their precursors were observed at day 2 that proceeded until day 30 following SCI. Many of these neurotransmitters were significantly decreased possibly reflecting neuronal cell death and decreased synthesis capacity after the injury.

**Figure 2:**
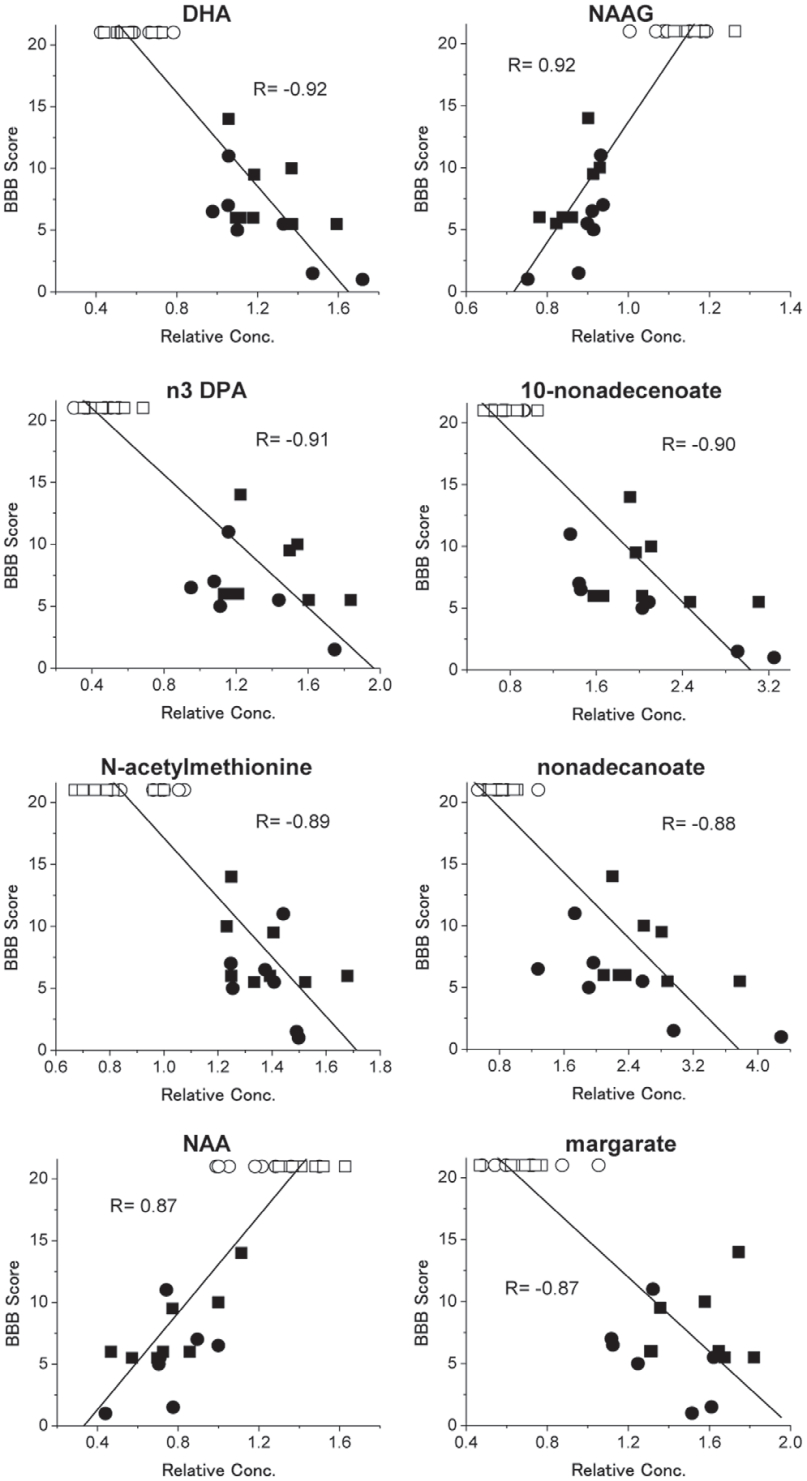
Correlation plots comparing BBB scores with metabolite levels at days 11 (circle) and 30 (square). The eight named metabolites with the highest |R| score are displayed (see Table S2 for complete list). Sham animals (day 11 and 30) received full BBB score ( = 21), consistent with normal movement. SCI at day 11 and 30 are depicted as •(n = 7) and ▪(n = 8). Sham at day 11 and 30 are displayed as ○(n = 8) and □(n = 8). All data points of Sham animals and SCI animals were used to calculate the R values.

In a secondary metabolic phase (day11 and day30), metabolites related to membrane remodeling or antioxidant defense were significantly altered (Table 1). Increased levels of the phospholipid degradation products glycerophosphocholine, lysolipids and fatty acids were observed. Several of the elevated fatty acids, including dihomo-linolenate, docosapentaenoate (DPA), and docosahexaenoate (DHA), are precursors to pro- and anti-inflammatory inflammation molecules. This biochemical signature is indicative of a dynamic inflammatory environment following SCI. Increased levels of ethanolamine and phosphoethanolamine, intermediates in phospholipid synthesis, implied that this pathway was also elevated from day 11. The activation of both pathways suggested that the inflammatory response following SCI removed debris from the injury and also initiated the repair process.

**Figure 3:**
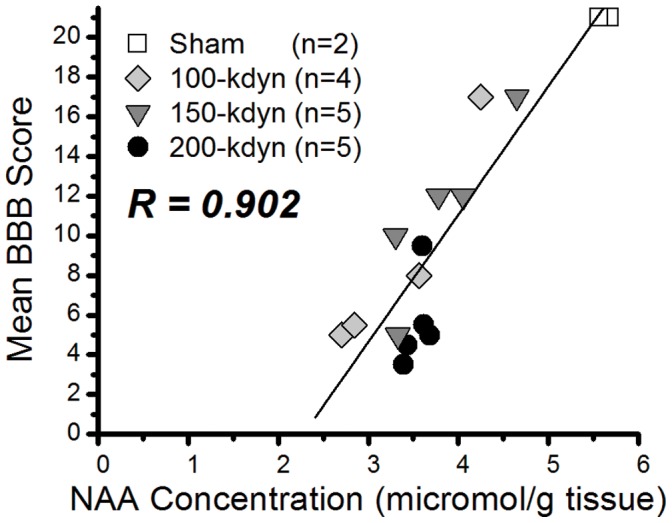
Correlation between absolute NAA concentration in the spinal cord and the corresponding BBB scores 32–34 days after SCI. SCI was induced by 100, 150 or 200-kdyn impact force. Sham animals received full BBB score ( = 21), consistent with normal movement.

Oxidative stress is thought to be a major contributor to the damage that occurs in the spinal cord following injury due to secondary effects. Increased levels of antioxidants such as ergothioneine, α-tocopherol, glutathione, and ascorbate were observed from day 11 following SCI suggesting that a broad spectrum of the defense system was mobilized.

### Metabolite Profiles Correlate Closely with BBB Scores

The 21-point open field locomotion scale developed by Basso, Beattie and Bresnahan (BBB) is widely used to assess locomotive recovery of SCI in rodents. BBB scores were measured after severe SCI (200-kdyn impact force) at days 11 and 30. Severe loss of locomotive activity was followed by limited spontaneous improvement in the BBB score over the 30 days, consistent with previous reports (Table S1) [7].

The distinct metabolic changes observed as a result of SCI raised the possibility that they may reflect the locomotive recovery process. We compared the metabolite levels to the BBB locomotive scores at both time-points. Many metabolites were observed to correlate greatly with the BBB score (Table S2). Among the top metabolites were compounds associated with the biochemical events following SCl, including neurotransmitter-related compounds and lipids (Figure 2). Decreased levels of *N-*acetyl-aspartyl-glutamate (NAAG) and *N-*acetyl-aspartate (NAA) resulted in positive correlations with the BBB score. In contrast, increased levels of several lipids including DHA and ω3-DPA (n3 DPA) showed highly negative correlations. These observations indicated that there was a biochemical basis for the BBB score suggesting that these metabolites may be potential chemical markers of locomotive recovery.

### Correlation of NAA with BBB Score

In order to illustrate that single metabolites can used to predict the locomotive score, we developed a GC-MS based assay to measure the absolute spinal cord concentrations of NAA in independent cohorts of mild to severe spinal cord injured rats at day 32–34. We focused on NAA because it is an abundant neurotransmitter-related compound that has been studied in SCI models and can be measured non-invasively by proton magnetic resonance spectroscopy (^1^H-MRS) [19], [20]. As illustrated in Figure 3, the NAA concentrations in spinal cord of injured animals were lower than those of Sham animals and correlated greatly with the BBB score (R>0.9). This result validated the findings from the metabolomics study and suggested that NAA could be used to predict the locomotive function.

## Discussion

In this study we successfully applied biochemical profiling to assess the global metabolic changes associated with SCI. This is, to our knowledge, the first study investigating global metabolomic fluctuations in the epicenter of the spinal cord after SCI. Dramatic metabolic changes were observed as a result of SCI. These changes were predominantly reflective of pathophysiological processes such as inflammation, tissue damage, clearance and remodeling after SCI, and consistent well with previous findings [4]–[6]. In addition, we found several metabolites, including NAA, NAAG, and the ω-3 fatty acids highly correlated with the established BBB score. Although Kwo et al. have reported potassium and calcium in spinal cord were correlated to injury severity [21], this is the first report, to our understanding, that shows the correlation between BBB scoring system; the index of neurobehavioral function, and metabolic changes in a rodent model. Moreover, we could find a biomarker candidate, NAA, which could be measured noninvasively in human and help to determine the degree of injury severity and to prognosticate neurologic recovery after SCI from this study.

Several microarray and proteomics studies were conducted to characterize the gene or protein expressions in spinal cord after experimental SCI in rats. *Carmel et al.* showed the neuronal loss and inflammatory response in gene expressions in the early phase [22]. *Resnick et al.* analyzed the delayed response in gene expressions and reported that the repair process was observed in spinal cord tissues [23]. *Yan et al.* investigated the time courses of protein expressions after the contusive SCI and reported the alterations of proteins associated with apoptosis, metabolism and cytoskeleton organization [24]. The changes in metabolites in spinal cord we demonstrated were also consistent with other omics profiles after SCI.

In the early metabolic phase, a complex picture of the inflammatory response emerged. The lipid mediators, PGE_2_ and PEA, were induced shortly after the injury. PGE_2_ has pro-inflammatory effects that may play a role in maintaining neuropathic pain [25], [26]. In contrast PEA has been shown to have anti-inflammatory properties indicating that a complex signaling network may have been activated to prevent an overactive and damaging inflammatory response [27]. Consistent with this notion, ω-6 and ω-3 polyunsaturated fatty acids, precursors of pro and anti-inflammatory lipid mediators, were increasingly elevated throughout the experiment suggesting that the inflammatory response was continuously being modulated. Activation of the inflammatory response is strongly associated with ROS formation. A broad spectrum of antioxidants was elevated consistent with mobilization of the defense system in response to the damage and secondary injury. These findings indicate that inflammation and oxidative stress pathways are active for a prolonged time period. It would thus be expected that they strongly contribute to the pathological outcome.

Neurotransmitters and related precursors (NAAG, NAA, aspartate and glutamate) remained suppressed following SCI. NAAG, NAA, and glutamate are abundant within neuronal cells, suggesting that the observed changes may reflect neuronal cell death [28]. Rapid release of excessive glutamate and other neurotransmitter related compounds that may contribute directly to cellular damage has been observed following SCI [29], [30]. The inability to restore these levels suggested that neuronal regeneration following SCI was limited.

We showed that many of the dramatic metabolic changes associated with SCI closely correlated with the established BBB locomotive score. The 21-point BBB scale represents a detailed and ordinal categorization of hindlimb locomotor recovery after spinal cord injury. In the BBB scoring system, joint movements are mainly assessed in the low score range (score 0–7), weight-supported plantar placements are evaluated in the middle score range (score 7–12) and coordination of movement is scored in the high score range (score 13–21) [7]. Metabolites that correlated best with the BBB scores were strongly associated with the biochemical events following SCl, including neurotransmitter-related compounds and lipids. The most abundant peptide neurotransmitter in the mammalian nervous system, NAAG, correlated highly with the BBB score. NAAG has been shown to be an important marker of neuronal viability. NAA levels are closely associated with neuronal function [31], and NAA concentration in spinal cord was decreased rapidly after the spinal cord injury in rats [32]. Decreased NAA levels have also been reported in various neurodegenerative diseases such as Alzheimer’s disease, amyotrophic lateral sclerosis and Parkinson’s disease [33]–[35]. *Blamire et al.* showed that decreases in NAA closely correlated with neuronal or axonal dysfunction loss in multiple sclerosis [36]. The NAAG and NAA levels were indicative of initial neuronal damage followed by limited regeneration, possibly reflecting impaired synthesis capacity. Several fatty acids correlated greatly with the BBB score. Many of these are important membrane constituents when incorporated into phospholipids. Their changes are reflective of damage and regeneration of the membrane. Increased levels of two abundant ω-3 fatty polyunsaturated acids (ω-3 PUFA), DHA and 5n3-DPA, showed strong negative correlations with the neurobehavioral function. These ω-3 PUFAs are specifically enriched in the brain and mainly anchored in the neuronal membrane as phospholipids, where it is involved in the maintenance of normal neurological function [37]. These increases in ω-3 PUFAs after SCI might reflect the cell damage in the spinal cord. On the other hand, recent evidence shows that ω-3 fatty acids or DHA can modulate several of the processes that contribute to secondary degeneration in the CNS and improve recovery from SCI [38], [39]. It is presumable that DHA increase in advance of SCI might promote neuroprotection by triggering the release of DHA-derived mediators, such as resolvins or neuroprotectin D1 [40]. In addition, multiple double bonds of ω-3 PUFAs are excellent targets for lipid peroxidation that could potentiate neurotoxicity, the increases of ω-3 PUFAs may also function as a free-radical scavenger to reduce the neuronal oxidative stress after SCI. The strong correlation with these metabolites suggests that the BBB score is closely related to the metabolic basis for cell damage, regeneration, and inflammatory response.

We developed a targeted assay to quantitatively measure NAA and conducted an additional experiment to demonstrate the utility of NAA measurement for the prediction of neurobehavioral function after SCI. High correlation with the BBB score was observed (Fig. 3) validating the findings from the metabolomics analysis. In this experiment, since many animals were severely injured and 6 of 14 showed almost same BBB scores (4.5–5.5), it was not clear whether the NAA concentrations in severely injured animals highly correlated with BBB scores. However, the overall correlation was very high (R>0.9), and a single metabolite, NAA, appears to correlate with and reflect what the locomotive score is like to be. *N-*acetyl-aspartate is a free amino acid, almost exclusively located in neurons and axons that can be measured non-invasively by ^1^H-magnetic resonance spectroscopy (^1^H-MRS) [19], [20]. Using ^1^H-MRS, *Qian* and his colleagues showed that NAA decreased rapidly after SCI and stayed depressed for 56 days in the epicenter as well as the rostral and caudal segments in rats, consistent with the findings in this study [20]. Our results suggest that the neurobehavioral function after SCI can be estimated by measuring the NAA concentration around the epicenter in spinal cord. Since NAA concentration in human spinal cord could be measured noninvasively by ^1^H-MRS [41], [42], NAA levels in spinal cord might provide a meaningful biomarker that could help to determine the degree of injury severity and to prognosticate neurologic recovery.

## Supporting Information

Table S1 **BBB score and body weight at day11 or day 30 after SCI induced by 200-kdyn impact force in metaboloimics study.** One of the outliers of PCA score plot (animal No. VK6, Figure 2) in SCI day11 group showed low BBB score.
(PDF)

Table S2 **Summary of correlation analysis comparing the BBB scores with analyte levels.**
(PDF)

Dataset S1 **Summary of metabolites with known chemical structures that show altered levels between SCI and Sham treated rats by Welch**’**s two sample t-test.** The values in bold font are statistically significant (p<0.05). The relative mean values for each group are included.
(XLS)
