# Supplementary material for: Metabolite Profiles Correlate Closely with Neurobehavioral Function in Experimental Spinal Cord Injury in Rats
Source: PLoS One. 2012 Aug 13;7(8):e43152. doi: 10.1371/journal.pone.0043152 (PMC3418274; doi:10.1371/journal.pone.0043152)
Supplement: Table S1 — BBB score and body weight at day11 or day 30 after SCI induced by 200-kdyn impact force in metaboloimics study. One of the outliers of PCA score plot (animal No. VK6, Figure 2) in SCI day11 group showed low BBB score. (PDF) [file pone.0043152.s001.pdf]

Table S1

BBB score and body weight at day11 or day 30 after SCI induced by 200-kdyn impact force in metabolimics study. One of the outliers of PCA score plot (animal No. VK6, Figure.2) in SCI day11 group showed low BBB score.

SCI, day11

Animal No.	BBB Score at day11		
	Left	Right	**Mean**
R3	4	6	**5.0**
R4	5	6	**5.5**
GR3	8	6	**7.0**
GR4	11	11	**11.0**
GR5	8	5	**6.5**
VK6	1	1	**1.0**
VR3	2	1	**1.5**
**MEAN**	5.6	5.1	**5.4**
**SE**	1.4	1.3	**3.4**

SCI, day30

Animal No.	BBB Score at day30		
	Left	Right	**Mean**
B2	6	6	**6.0**
B3	6	5	**5.5**
GB2	11	9	**10.0**
GB3	6	5	**5.5**
VR4	6	6	**6.0**
VR9	6	6	**6.0**
VB1	14	14	**14.0**
VB6	10	9	**9.5**
**MEAN**	8.1	7.5	**7.8**
**SE**	3.1	3.1	**3.1**
