# Supplementary material for: Metabolite Profiles Correlate Closely with Neurobehavioral Function in Experimental Spinal Cord Injury in Rats
Source: PLoS One. 2012 Aug 13;7(8):e43152. doi: 10.1371/journal.pone.0043152 (PMC3418274; doi:10.1371/journal.pone.0043152)
Supplement: Table S2 — Summary of correlation analysis comparing the BBB scores with analyte levels. (PDF) [file pone.0043152.s002.pdf]

Table S2

Summary of correlation analysis comparing the BBB scores with analyte levels.

Metabolite	correlation coefficient (R)	∣R∣
docosahexaenoate (DHA; 22:6n3)	-0.92	0.92
N- acetyl-aspartyl-glutamate (NAAG)	0.92	0.92
docosapentaenoate (n3 DPA; 22:5n3)	-0.91	0.91
10-nonadecenoate (19:1n9)	-0.90	0.90
N- acetyl-methionine	-0.89	0.89
nonadecanoate (19:0)	-0.88	0.88
X - 12854	-0.88	0.88
X - 13418	-0.87	0.87
N- acetyl-aspartate (NAA)	0.87	0.87
margarate (17:0)	-0.87	0.87
tyrosine	-0.86	0.86
mannose	-0.86	0.86
sphingosine	-0.86	0.86
valine	-0.86	0.86
phosphoethanolamine	-0.85	0.85
docosatrienoate (22:3n3)	-0.85	0.85
eicosapentaenoate (EPA; 20:5n3)	-0.84	0.84
1-oleoylglycerophosphoserine	-0.84	0.84
docosadienoate (22:2n6)	-0.84	0.84
guanosine	0.84	0.84
N- acetyl-glutamate	0.84	0.84
dihomo-linolenate (20:3n3 or n6)	-0.83	0.83
adrenate (22:4n6)	-0.83	0.83
hypotaurine	-0.83	0.83
dihomo-linoleate (20:2n6)	-0.83	0.83
X - 15163	-0.82	0.82
galactose	-0.82	0.82
gamma-glutamylleucine	-0.82	0.82
lysine	-0.82	0.82
leucine	-0.82	0.82
glucose	-0.81	0.81
docosapentaenoate (n6 DPA; 22:5n6)	-0.81	0.81
X - 12748_201	-0.81	0.81
xanthosine	-0.81	0.81
glutamate	0.80	0.80
X - 12627	-0.80	0.80

X-xxxxx refers to unnamed metabolite.
